# Applications, Challenges, and Prospects of Artificial Intelligence in Crop Production

**DOI:** 10.3390/plants15121863

**Published:** 2026-06-16

**Authors:** Congshan Xu, Ruirui Chen, Xiaodong Huang, Yi Han, Ning Tong, Shuanghong Shen

**Affiliations:** 1 Anhui Product Quality Supervision & Inspection Research Institute (National Centre for Inspection & Testing of Drainage Irrigation & Water-Saving Equipment Products Quality), Hefei 230051, China; xucongshan1027@163.com (C.X.);; 2 Department of Biology, Anhui Agricultural University, Hefei 230031, China; 3 Anhui Science and Technology Achievement Transformation Promotion Center (Anhui Provincial Institute of Science and Technology), Hefei 230001, China; 4 Institute of Artificial Intelligence, Hefei Comprehensive National Science Center, Hefei 230031, China

**Keywords:** artificial intelligence, crop production, biotic stress monitoring, soil health, supply chain optimization

## Abstract

With the growing global population, intensifying resource constraints, and deepening climate change impacts, agriculture faces dual challenges of ensuring food security and advancing sustainable development. Artificial intelligence (AI) has emerged as a transformative technology, penetrating the entire crop production chain and offering innovative solutions to traditional agricultural bottlenecks. This paper systematically reviews AI applications in five core domains: biotic stress monitoring, soil health management, precision operation, supply chain optimization, and climate-resilient agriculture. It further categorizes and analyzes four key technical pathways—deep learning, sensor fusion, data-driven methods, and hybrid modeling—while critically examining major challenges across data, technology, implementation, and ethics/policy dimensions. Future directions are discussed from technological innovation, scenario expansion, implementation guarantees, and sustainability orientation. Research findings show that AI has achieved technical validation in pest/disease detection, soil parameter modeling, and intelligent spraying, with accuracy exceeding 85% in some cases. However, regional data bias, insufficient model generalization, and the digital divide still hinder large-scale deployment. Moving forward, coordinated efforts in technological innovation and policy support are required to promote inclusive, standardized, and sustainable AI applications in crop production.

## 1. Introduction

As a fundamental industry ensuring human survival, agriculture is facing multiple pressures, including the expansion of food demand caused by population growth, ecological degradation due to over-exploitation of resources, and disaster risks induced by climate change [1] According to statistics from the Food and Agriculture Organization of the United Nations (FAO), biotic stresses cause an annual global crop yield reduction of 20–40%, with economic losses exceeding 220 billion US dollars [2]; meanwhile, problems such as soil degradation and water scarcity further exacerbate the uncertainty of agricultural production. Traditional agriculture relies on empirical decision-making, with limitations such as inefficient resource utilization and passive risk response, which can no longer meet the needs of modern agricultural development.

Artificial intelligence, with its powerful data processing, pattern recognition, and prediction capabilities, has provided a new technical paradigm for agricultural transformation. From field production to supply chain management, AI technology, through integration with remote sensing, the Internet of Things (IoT), and robotics, has realized the precision, automation, and intelligence of agricultural production [3]. In recent years, the global agricultural AI market has continued to expand, with a projected compound annual growth rate of 25.5% from 2020 to 2026 [1], and relevant research results have continuously emerged in scenarios such as pest and disease detection, soil parameter modeling, and intelligent spraying.

Based on 69 core studies and derived secondary references, this paper constructs an analytical framework of “application scenarios-technical pathways-existing challenges-future prospects”, systematically integrates the research results and practical experience of AI in the agricultural field, aiming to provide references for relevant research and policy formulation.

## 2. Application Scenarios of Artificial Intelligence in Agriculture

### 2.1. Biotic Stress Monitoring

**Figure 1:**
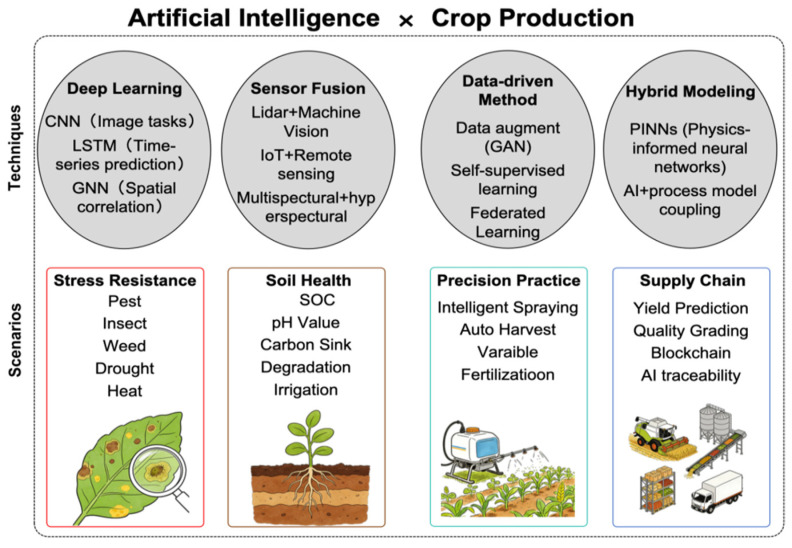
Artificial Intelligence Application Scenarios and Core Techniques in Crop Production.

Biotic stresses (pests, diseases, pathogenic infections, etc.) are the primary factors restricting crop yields. Through means such as image recognition and spectral analysis, AI technology has realized the early detection, precise classification, and severity assessment of stresses, which is significantly superior to traditional visual observation and laboratory detection methods (Figure 1).

Recent advances in multi-sensor integration and artificial intelligence (AI) have significantly promoted crop phenotyping and stress detection under complex field conditions [4]. Deep learning, especially convolutional neural networks (CNNs), has become the mainstream approach for disease and pest identification, achieving reliable performance in diverse crop scenarios [5]. Unmanned aerial vehicle (UAV) multi-modal imaging, combined with ground sensors, provides multi-scale phenotypic data and improves cross-environment generalization [6]. Lightweight architectures, such as MobileNet and YOLO, enable real-time inference on edge devices for field deployment [7] Self-supervised learning effectively mitigates labeled data scarcity and enhances model adaptability [8]. Hyperspectral imaging facilitates early detection of subtle stress symptoms invisible to the naked eye [9]. Three-dimensional (3D) reconstruction supports automated harvesting and precision management by capturing plant structural traits [10]. Transfer learning and domain adaptation further address cross-crop and cross-region distribution shifts [11]. Large-scale datasets, including PlantVillage, IP102, and DLCPD-25, provide diverse benchmarks for robust model training [12]. Deep learning achieves high accuracy in paddy disease detection [13]. Fuzzy systems support rice disease diagnosis [7]. In addition to image data, AI systems integrating meteorological data and field sensor data have also shown potential.

In terms of disease detection, deep learning models have become the core technical support. Convolutional Neural Networks (CNNs), with their strong local feature extraction capabilities, have performed prominently in controlled-environment disease detection (e.g., rice blast, bacterial leaf streak), achieving a high accuracy of 92–99.75% [2]. However, CNNs lack global context perception and struggle with small pests or complex field backgrounds, limiting real-field generalization. By contrast, YOLO-based models excel in real-time field detection but sacrifice accuracy for tiny targets [13]; Vision Transformers (ViTs) handle subtle stress symptoms better but demand high computing resources [2]. Transfer learning reduces data reliance but faces cross-domain shift issues [14]. Beyond single-model applications, AI systems integrating meteorological and field sensor data further improve monitoring robustness. The deep CNN model proposed by [5], combined with the ResNet-V2 network architecture, further improved the detection accuracy and stability. In addition to image data, AI systems integrating meteorological data and field sensor data have also shown potential. For example, the AgriTalk platform realizes real-time early warning of rice blast by integrating micro-meteorological data such as temperature and humidity with AI models [15].

In the field of pest monitoring, AI technology has achieved a leap from manual counting to automated recognition. Nasser Shah et al. (2019) adopted the K-means clustering algorithm and image processing technology to construct a brown planthopper recognition and counting system, solving the problems of low efficiency and large errors in traditional sticky trap monitoring [16]. Xu et al., 2025 developed a pest detection model based on CNN, which improved the pest recognition ability in complex backgrounds through RGB and HSI color space conversion [17].

In addition, the combination of Unmanned Aerial Vehicles (UAVs) and AI has expanded the monitoring range. Wang et al. (2025) used UAVs equipped with multispectral cameras and combined with the YOLOv5 algorithm to achieve high-speed detection of pest-infested plants, with an accuracy of 97.3% [18].

Unmanned aerial vehicle (UAV) multi-modal imaging, combined with ground sensors, provides multi-scale phenotypic data and improves cross-environment generalization [6,8]. Lightweight architectures, such as MobileNet and YOLO, enable real-time inference on edge devices for field deployment [7]. Self-supervised learning effectively mitigates labeled data scarcity and enhances model adaptability [8,9]. Hyperspectral imaging facilitates early detection of subtle stress symptoms invisible to the naked eye [9]. Three-dimensional (3D) reconstruction supports automated harvesting and precision management by capturing plant structural traits [10]. Transfer learning and domain adaptation further address cross-crop and cross-region distribution shifts [11]. Large-scale datasets, including PlantVillage, IP102, and DLCPD-25, provide diverse benchmarks for robust model training [12].

In terms of weed identification and control, the collaborative application of sensor fusion and AI classification models has achieved remarkable results. Partel et al. (2019) developed a low-cost intelligent weed management system, which realized the precise distinction between rice and weeds through machine vision and AI algorithms, providing decision support for variable rate spraying [7]. Sen Debleena and Barnwal (2020) proposed a transfer learning method, which improved the robustness of weed classification models in different field environments, with a detection accuracy of 85–99% [19]. RGB, multispectral, and hyperspectral sensors equipped on UAVs provide multi-source data for weed monitoring. Among them, hyperspectral sensors have shown unique advantages in early weed identification due to their fine spectral resolution [20]. Advanced weed segmentation datasets further boost model performance [21].

The assessment of biotic stress severity is an extended direction of AI application. Patil et al. (2023) adopted EfficientNet-B0 and RCNN models to achieve a quantitative assessment of rice disease severity by labeling disease areas and calculating the proportion of infected area [22]. Paauw et al., 2024 used VCC labeling and Make Sense tools to accurately locate leaf infection areas, providing technical support for severity grading [23].

### 2.2. Climate-Resilient Agriculture

Facing challenges such as frequent extreme weather events and variations in growth environments caused by climate change, AI technology improves the climate resilience of agricultural systems through applications such as disaster early warning and adaptive planting.

In terms of disaster early warning, AI models realize precise risk prediction by integrating multi-source data. Pham et al. (2024) adopted a data-driven method, combining satellite data and meteorological observations to construct a high-precision spatiotemporal precipitation estimation model, providing support for flood disaster early warning [24]. The IBM Watson decision platform realizes early warning of disasters such as drought and hail through AI algorithms analyzing meteorological data and crop growth data [25]. In pest and disease disaster early warning, AI models realize early prediction of outbreak risks by analyzing climate conditions and historical pest and disease occurrence data [2].

In terms of adaptive planting, AI technology provides a scientific basis for crop variety selection and planting system adjustment. Platforms such as Digital Green recommend drought-resistant, waterlogging-tolerant, and other adaptive crop varieties to smallholder farmers through AI algorithms analyzing regional climate characteristics and soil conditions [26]; Liu et al. (2022) adopted ANN models combined with GDD data to optimize crop planting cycles under different climate scenarios [27]. Soil water capacity mapping supports climate-resilient planning [8]. In addition, AI models provide decision support for the adjustment of planting structures by simulating the impact of climate change on crop growth [17].

### 2.3. Soil Health Management

As the basic carrier of agricultural production, soil health directly affects crop growth and ecological sustainability. Through the integration of multi-source data, AI technology realizes soil parameter modeling, carbon sink estimation, and degradation monitoring, providing scientific tools for the precise management of soil resources.

In terms of soil parameter modeling, machine learning models have been widely used in the prediction of key soil parameters such as Soil Organic Carbon (SOC), pH value, and texture. Random Forest (RF) has become the most widely used model due to its advantages, such as anti-overfitting and feature importance evaluation, and has shown better performance than traditional geostatistical methods in the prediction of SOC spatial distribution [28]. Support Vector Machines (SVM) and Neural Networks (NN) have also been widely applied in soil parameter modeling. For example, Liu et al. (2022) adopted Artificial Neural Networks (ANN) combined with Growing Degree Days (GDD) to construct a correlation model between rice growth and soil parameters [27]. Yüzügüllü et al. (2024) used SVM combined with remote sensing data to achieve precise mapping of soil texture and pH value [29]. In addition, hybrid modeling methods such as Physics-Informed Neural Networks (PINNs) improve the physical consistency of parameter prediction by integrating soil process mechanisms with data-driven models [3].

Carbon sink estimation and Soil Organic Matter (SOM) monitoring are important applications of AI in the field of soil ecology. Dong et al. (2025) revealed the correlation between crop residue coverage and SOC accumulation through interpretable machine learning models, improving the accuracy of carbon sink estimation [30]. Zhang et al. (2025) constructed a cumulative crop residue index using time-series remote sensing data, optimizing the monitoring effect of SOM in black soil areas [12]. Hybrid modeling improves physical consistency in SOC prediction [30]. These studies provide technical support for the realization of agricultural carbon neutrality goals.

In terms of soil degradation monitoring, AI technology has realized the precise identification of degradation types such as soil erosion, salinization, and compaction. Raj et al. (2024) completed soil erodibility mapping in the Himalayan region using RF models combined with remote sensing and field data [31]; Naimi et al. (2021) constructed a spatial distribution prediction model of soil salinization by integrating environmental covariates using machine learning methods [32]. Schweng et al. (2026) pointed out that AI models can realize graded assessment of degradation degree by analyzing the synergistic changes in physical, chemical, and biological indicators of soil, providing a decision-making basis for soil remediation [3]. Machine learning integrates covariates to map soil salinity [13].

### 2.4. Precision Operation

Precision operation is the core link of smart agriculture. Through the integration with automated equipment, AI technology has realized the precision and efficiency of operations such as intelligent spraying, automated harvesting, and variable rate fertilization, significantly reducing resource waste and labor intensity. AI-driven precision systems have been widely applied in intelligent spraying, mechanical weeding, and autonomous harvesting [1]. Deep learning integrated with oscillating pneumatic mechanisms enables accurate intra-row weeding in lettuce under variable field conditions [27]. Visual navigation combining machine vision and LiDAR supports autonomous robot operation in both open fields and protected environments [33]. Data-driven decision-making and hybrid modeling further optimize resource allocation efficiency [3].

In the field of intelligent spraying, the application of sensor fusion and AI decision-making systems has achieved remarkable results. Partel et al. (2021) developed a low-cost intelligent sprayer, integrating LiDAR, machine vision, and GPS technologies, realizing tree classification and fruit counting through CNN models, and dynamically adjusting the spraying amount according to target crop characteristics, reducing chemical agent usage by 28% [7]; Mahmud et al. (2021) developed a LiDAR-based canopy density measurement system, generating spraying maps through cloud processing to provide precise guidance for variable rate spraying [34]. In addition, the combination of UAV spraying and AI has expanded the operation range. Nanavati et al. (2023) proposed a data-driven path planning framework to optimize the uniform coverage effect of UAV spraying [35]. Sensor-based autonomous spraying improves pesticide efficiency [27]. In addition, the combination of UAV spraying and AI has expanded the operation range. Automatic spraying adaptation reduces pesticide waste in orchards [10]. 

In terms of automated harvesting, significant progress has been made in the integration of computer vision and robotics. Manta-Costa et al. (2024) adopted machine vision and deep learning models to realize precise monitoring of pecan fruit growth status, providing support for harvesting time decision-making [36]; Sparrow and Howard (2021) developed a fruit harvesting robot, which identifies mature fruits through CNN models to achieve selective harvesting, reducing harvesting losses [37]. In the harvesting of grain crops such as rice and wheat, autonomous tractors combined with AI path planning technology have improved operation efficiency and reduced field compaction [1].

Variable rate fertilization and irrigation are another important application of AI in precision operations. Ekanayake et al. (2023) developed an AI optimization model to dynamically adjust fertilizer formulas according to the nutritional needs of hydroponic crops [38]; intelligent irrigation systems such as NetBeat realize precise monitoring of soil moisture and dynamic optimization of irrigation volume through IoT sensor data and AI algorithms [38]. In variable rate fertilization, RF and XGBoost models generate field fertilization prescription maps by analyzing data such as soil parameters and crop growth status, improving fertilizer use efficiency [3].

### 2.5. Supply Chain Optimization

By integrating data from the entire chain of production, circulation, and consumption, AI technology realizes the intelligence of yield prediction, quality grading, and logistics scheduling, providing a guarantee for the efficient operation of the agricultural supply chain.

In terms of yield prediction, the application of multi-source data fusion and machine learning models has improved prediction accuracy. Vijayakumar et al. (2021) combined ground fruit detection data with UAV images to realize citrus yield prediction through AI models [39]; Chew et al. (2020) adopted deep transfer learning methods to construct yield prediction models based on crop growth period image data, which showed good adaptability in crops such as rice and wheat [40]. In addition, time-series models such as LSTM have improved the reliability of long-term yield prediction by analyzing time-series information such as historical yields and meteorological data [3].

In the field of quality grading, computer vision and AI classification models have become core technologies. Bhargava and Bansal (2018) reviewed the application of machine vision in fruit and vegetable quality grading, pointing out that CNN models can achieve precise grading based on features such as color, shape, and texture [41]; Costa et al. (2021) developed a pecan quality grading system, which extracts features through machine vision and combines deep learning models to realize automatic determination of quality grades [42]. In grain quality grading, the combination of hyperspectral imaging and AI models has realized rapid detection of internal quality indicators such as protein content and moisture [9].

In terms of logistics scheduling optimization, AI algorithms reduce supply chain costs and losses by optimizing transportation routes and inventory management. Ryan et al. (2023) proposed an AI-driven logistics optimization model, which realizes dynamic adjustment of agricultural product transportation routes by combining real-time traffic, market demand, and other data [43]; platforms such as AgroCenta help smallholder farmers optimize sales decisions and reduce losses in circulation links through AI price prediction and supply-demand matching algorithms [44]. The integration of blockchain and AI further improves supply chain transparency, realizing full traceability of agricultural products from the field to the table.

## 3. Key Technical Pathways of Artificial Intelligence in Agricultural Applications

Deep learning, machine learning, and hybrid models constitute the core technical framework for agricultural AI [15]. CNNs and transformers dominate image classification and detection tasks [2]. Recurrent neural networks (RNNs) and graph neural networks (GNNs) are widely used for time-series prediction and spatial correlation modeling [3]. Sensor fusion, data augmentation, and physics-informed learning enhance prediction accuracy and interpretability [36].

### 3.1. Deep Learning

As a core branch of AI technology, deep learning has been widely applied in the agricultural field due to its strong capabilities of automatic feature extraction and complex pattern recognition, mainly including model architectures such as CNN, LSTM, and GNN.

CNNs dominate image-related tasks, especially in scenarios such as pest and disease detection and crop growth monitoring. In addition to traditional CNN models (VGG, ResNet, etc.), lightweight models (MobileNet, EfficientNet) are increasingly used in real-time field detection due to their adaptability to embedded devices. For example, Anami et al. (2020) adopted the MobileNet model to construct a rice stress recognition system, achieving efficient operation on mobile devices [45]; Partel et al. (2021) adopted the YOLOv3 framework, combined with ResNet50 and Darknet53 backbone networks, to realize tree classification and fruit counting, respectively, balancing detection accuracy and speed [7].

Time-series models such as LSTM have unique advantages in processing time-series data, mainly used in scenarios such as yield prediction and meteorological disaster early warning. Wu et al. (2023) realized time-series prediction of soil temperature and humidity based on LSTM models combined with IoT sensor data [46]; Ning et al. (2022) compared the performance of ARIMA, LSTM, and Prophet models in crop yield prediction, and found that LSTM models have more advantages in capturing long-term trends and seasonal changes [47].

As an emerging model architecture, GNN has shown potential in scenarios such as spatial prediction of soil parameters and simulation of crop pest and disease transmission by modeling spatial correlation relationships. Zha et al. (2024) adopted GNN models to predict soil heavy metal pollution in the Pearl River Basin, improving prediction accuracy by capturing spatial dependencies [48]; Schweng et al. (2026) pointed out that GNN has unique advantages in addressing soil spatial autocorrelation issues, but its application in the agricultural field is still in the initial stage [3].

### 3.2. Sensor Fusion

Sensor fusion technology improves the input quality and robustness of AI models by integrating multi-type and multi-source sensor data, mainly including fusion modes such as LiDAR + machine vision and IoT + remote sensing.

The fusion of LiDAR and machine vision is widely used in crop structural parameter extraction and target recognition. The intelligent sprayer system developed by Partel et al. (2019) integrates LiDAR and USB camera data [7]. LiDAR is used to measure tree height and canopy density, and machine vision is used for tree classification and fruit detection, realizing multi-dimensional data support for spraying decisions [7]; Cheein et al. (2015) adopted LiDAR and visual sensor fusion to realize real-time reconstruction of the 3D structure of orchard canopies [49]. This fusion mode makes up for the limitations of a single sensor: LiDAR provides precise spatial information, and machine vision provides rich texture and color information [50,51].

The fusion of IoT and remote sensing realizes comprehensive monitoring from the field point scale to the regional surface scale. The intelligent agricultural system developed by Sharma et al. (2025) integrates field IoT sensors and satellite remote sensing data to realize multi-scale monitoring of crop growth [26]; the RiceTalk platform developed by Chen collects field micro-meteorological data through IoT sensors and combines remote sensing images to realize precise early warning of rice blast [15]. In addition, UAV remote sensing, as a medium-scale data source connecting IoT and satellite remote sensing, has become a research hotspot in fusion with ground sensor data [52].

The fusion of multispectral and hyperspectral sensors further expands the data dimension. Sulaiman et al. (2022) reviewed the application of hyperspectral remote sensing in rice weed detection, pointing out that the fine spectral features of hyperspectral data can improve the distinguishability between weeds and crops [20]; Abdulridha et al. (2020) adopted the fusion of hyperspectral imaging and machine learning models to realize early detection of tomato diseases, with higher accuracy than detection methods based on RGB images [52].

### 3.3. Data-Driven Methods

Data-driven methods provide a guarantee for the performance improvement of AI models by optimizing data quality and utilization efficiency, mainly including technologies such as data augmentation, self-supervised learning, and federated learning.

Data augmentation technology effectively solves the problem of scarce labeled data in the agricultural field. Divyanth et al. (2022) adopted an image-to-image conversion enhancement method to improve the generalization of crop/weed classification models [53]; Madsen et al. (2019) used Generative Adversarial Networks (GANs) to generate artificial images of plant seedlings, expanding the training dataset [54]; Di Cicco et al. (2017) randomized environmental features through a graphics engine to generate synthetic crop and weed images, reducing data collection costs [55]. GAN-based transfer learning further optimizes weed identification [55]. These methods reduce the risk of model overfitting by increasing data diversity [56].

Self-supervised learning and semi-supervised learning reduce the dependence on labeled data by utilizing a large amount of unlabeled data. Epifani et al. (2023) adopted a self-supervised semantic segmentation method to realize automatic distinction between olive trees and vineyards without manual labeling [8]; Lad and Raval (2022) proposed a data-centric method to improve the generalization of wheat spike detection models using unlabeled data through domain variance reduction [57].

As a privacy-preserving AI technology, federated learning is of great significance in cross-regional agricultural data sharing. Schweng et al. (2026) pointed out that federated learning can realize collaborative modeling of farmer data in different regions, avoiding the leakage of sensitive data [3]; Zhang et al. (2024) reviewed the application prospects of federated learning in agriculture, believing that it has potential in addressing regional data bias and improving the global adaptability of models [58].

### 3.4. Hybrid Modeling

Hybrid modeling combines data-driven models with physical mechanism models, taking into account both prediction accuracy and physical rationality, mainly including modes such as Physics-Informed Neural Networks (PINNs) and AI + process model coupling.

PINNs integrate physical laws as constraint conditions into neural network training, and their applications in scenarios such as soil moisture movement simulation and crop growth prediction are gradually increasing. Cuomo et al. (2025) adopted PINNs to predict soil microbial growth, improving the physical consistency of prediction by integrating microbial metabolic mechanisms [59]; Schweng et al. (2026) pointed out that PINNs are more interpretable and generalizable than pure data-driven models when dealing with problems with clear physical processes, such as soil parameter prediction [3].

The coupling of AI and process models realizes complementary advantages. Hu et al. (2023) combined physical models with PINNs to optimize energy-efficient food production [21]; Liu et al. (2024) combined crop growth process models with machine learning algorithms to predict actual evapotranspiration of maize at the regional scale [60]. This coupling mode not only utilizes the physical basis of process models but also exerts the advantages of AI models in processing complex nonlinear relationships [3] (Table 1).

## 4. Existing Challenges in the Application of Artificial Intelligence in Agriculture

Data scarcity, domain shift, class imbalance, and insufficient diversity remain major bottlenecks limiting real-world deployment [44]. Most existing datasets are collected under controlled conditions, lacking complex field variations [12]. The absence of large-scale, long-tailed datasets hinders generalizable model development [60]. Deficiencies in model interpretability and edge deployment capability further restrict practical applications [3].

### 4.1. Data-Level Challenges

Data is the foundation for AI model training. Currently, the application of agricultural AI faces prominent problems such as regional bias, uneven quality, and a lack of standardization.

Regional data bias leads to insufficient model generalization. Schweng et al. (2026) found through analyzing global soil AI research data that data in tropical, arid, temperate continental, and polar tundra climate zones is seriously insufficient, while these regions often have important agricultural value [3]; research by Benfenati et al. (2025) showed that agricultural AI research data is mainly concentrated in regions such as China, India, Europe, and the United States, with scarce data in underdeveloped regions such as Africa and South America [56]. This regional imbalance leads to a significant decline in model performance in data-scarce regions [57]. The quality–quantity trade-off limits pest recognition robustness [59].

Uneven data quality affects model performance. Agricultural data collection is affected by various factors such as environmental conditions, sensor accuracy, and operating specifications, resulting in problems such as high noise, missing values, and labeling errors [56]. For example, field image data is easily disturbed by light, weather, soil background, etc., and soil sampling data may have errors due to non-standard sampling methods [3]. In addition, the data formats and labeling standards adopted by different research teams are inconsistent, making it difficult to share and integrate data.

The lack of data standardization restricts industry development. Currently, there is a lack of unified standards for data collection, labeling, storage, and sharing in the agricultural AI field. Data formats of different sensors are incompatible, and labeling rules of different studies are inconsistent, leading to difficulties in cross-study and cross-regional data integration [56]. For example, there are differences in disease classification systems and severity grading standards in pest and disease datasets, making it difficult to carry out model training and performance comparison [2].

### 4.2. Technical-Level Challenges

The combination of the characteristics of AI technology itself and the complexity of agricultural scenarios leads to challenges in model generalization, lightweight deployment, and interpretability.

Insufficient model generalization makes it difficult to adapt to complex agricultural environments. Agricultural production is affected by various factors such as climate, soil, and crop varieties, with strong scene heterogeneity. Existing AI models are mostly trained in specific regions, specific crops, and specific environmental conditions, and their performance often declines significantly when applied to new scenarios [1]. For example, a rice disease detection model trained in one region may have reduced accuracy in another region due to variety differences and disease symptom variations [2]; the migration performance of soil parameter prediction models in regions with different soil types is limited [3].

Difficulties in lightweight deployment restrict real-time field applications. Most field agricultural applications rely on platforms with limited computing power, such as embedded devices, UAVs, and mobile terminals, while complex AI models (such as deep CNNs and GNNs) have high computational costs and cannot meet real-time requirements [60]. Although lightweight models such as MobileNet and YOLOv3-Tiny have made certain progress, the balance between accuracy and speed still needs to be optimized. In addition, model deployment requires professional and technical personnel, increasing the application threshold.

Insufficient model interpretability affects user trust. Most AI models (especially deep learning models) are regarded as “black boxes”, and their decision-making processes are difficult to explain, leading to farmers’ and decision-makers’ lack of trust in model outputs [3]. For example, pest and disease detection models only output detection results and cannot explain why they are judged as such diseases, making it difficult to assist farmers in understanding the causes of disease occurrence; the outputs of soil parameter prediction models lack analysis of key influencing factors, limiting the promotion and application of models [54]. Although interpretable AI technologies such as SHAP and LIME are gradually being applied, their popularity in the agricultural field is still low [43].

In addition to the above technical challenges, the current discussion of large language models (LLMs), model interpretability, and policy-level issues remains overly general and lacks specific, actionable research gaps. For agricultural LLMs, existing studies primarily focus on general-purpose models rather than crop-specific, domain-adapted LLMs fine-tuned with agronomic knowledge, field phenotyping data, and regional farming practices. There is a critical gap in integrating LLMs with multimodal agricultural data (e.g., images, spectra, sensor readings) to support end-to-end crop stress diagnosis and decision-making. Furthermore, cross-lingual and cross-regional generalization of agricultural LLMs is severely limited, especially for smallholder farming contexts with fragmented data. Regarding model interpretability, current agricultural AI research rarely establishes standardized evaluation frameworks tailored to crop production scenarios. While methods such as SHAP and LIME have been widely discussed, their practical deployment in real-world field models remains limited. There is a notable lack of farmer-friendly interpretability tools that can visualize model decisions in intuitive agronomic terms, which hinders trust and adoption among end-users.

### 4.3. Implementation-Level Challenges

The transformation of AI technology from the laboratory to the field faces practical obstacles such as the digital divide, adaptability to smallholder farmers, and maintenance costs.

The digital divide exacerbates agricultural inequality. There are significant gaps in digital infrastructure and technology access capabilities between developed and underdeveloped regions. In regions such as Africa and South America, low Internet coverage and unstable power supply in rural areas restrict the operation of IoT devices and AI systems [1,3,60]; smallholder farmers, due to limited economic conditions, cannot afford the purchase cost of AI equipment [34]. In addition, insufficient digital literacy is also an important constraint factor, as many farmers lack the ability to operate AI equipment and interpret model outputs [61].

Insufficient adaptability to smallholder farmers limits large-scale application. Global agricultural production is dominated by smallholder farmers, but their production scale is small, plots are scattered, and resources are limited. Existing AI technologies are mostly designed for large-scale commercial farms and cannot meet the needs of smallholder farmers [61]. For example, large-scale intelligent spraying equipment is expensive and unaffordable for smallholder farmers; complex AI decision-making systems require professional knowledge, which is mismatched with the actual operational capabilities of smallholder farmers [7]. In addition, the small amount of production data of smallholder farmers makes it difficult to support the training of personalized AI models [2,3].

High maintenance costs affect technical sustainability. The maintenance of AI agricultural equipment requires professional and technical personnel and continuous capital investment, including sensor calibration, model updates, and equipment maintenance [1]. In rural areas, the shortage of professional and technical personnel makes it difficult to repair equipment in a timely manner after failures; model updates require continuous data support and algorithm optimization, increasing long-term use costs [62]. In addition, some AI systems rely on cloud services, and continuous network fees also become a burden for smallholder farmers.

### 4.4. Ethical and Policy-Level Challenges

The application of AI in the agricultural field has triggered ethical and policy issues such as data privacy, algorithmic fairness, and imperfect regulatory systems.

Data privacy and security risks are prominent. Agricultural data includes sensitive content such as farmers’ production information, plot locations, and soil properties. Improper management may lead to privacy leakage and data abuse [1,3]. For example, farmer data collected by large agricultural technology companies may be used for commercial competition, harming farmers’ interests; the risk of data leakage increases during cross-regional data sharing [63]. Currently, many countries lack privacy protection laws for agricultural data, and the definition of data ownership and usage rights is unclear [3,4].

Algorithmic fairness issues arouse social concerns [53]. The training data of AI models may have biases, leading to unfair algorithm outputs for specific groups. For example, models trained based on data from developed regions may produce discriminatory results when applied in underdeveloped regions; AI systems targeting specific crop varieties may ignore the needs of growers of niche varieties [1]. In addition, the application of AI technology may exacerbate the polarization of agricultural production: large farms obtain more technical dividends due to resource advantages, while smallholder farmers face the risk of marginalization [60].

The imperfect regulatory system lags behind technological development. AI agricultural technology updates rapidly, while the formulation of relevant regulatory policies and standards is relatively lagging. Currently, there are no unified standards for the performance of AI agricultural equipment, model safety, and data usage specifications, leading to uneven product quality in the market [1]. For example, there are no unified evaluation standards for the spraying accuracy of intelligent spraying equipment and the accuracy of AI disease detection models, making it difficult to protect user rights and interests; the liability definition for AI algorithms is unclear, and when model decisions lead to losses, it is difficult to determine the liability [4].

## 5. Future Prospects

Artificial intelligence has demonstrated great potential in crop production, covering biotic stress monitoring, soil health management, precision operation, supply chain optimization, and climate-resilient agriculture. However, challenges such as data scarcity, insufficient generalization, and limited field deployment still restrict large-scale applications. Future research can be systematically divided into short-term technical improvements, long-term research opportunities, and practical implementation challenges, with a focus on integrating foundation models, large-scale agricultural datasets, multimodal phenotyping platforms, domain adaptation, and lightweight edge-AI systems into crop production.

### 5.1. Short-Term Technical Improvements

In the short term, efforts should focus on addressing immediate technical bottlenecks and enhancing the practicality of current AI systems. Lightweight and interpretable AI techniques will be prioritized for edge deployment, while multimodal fusion and small-sample learning will improve performance under complex field conditions.

Lightweight and edge-deployable AI systems are essential for real-time field applications. Current deep learning models, including CNNs and transformers, often have high computational costs, limiting their use on UAVs, ground robots, and portable devices. Short-term work should optimize lightweight architectures such as MobileNet, YOLOv3-Tiny, and compressed ViT variants, combining pruning, quantization, and knowledge distillation to balance accuracy and speed [53,64]. These lightweight models can be directly deployed for real-time disease and pest detection in field environments.

Multimodal data fusion will further enhance monitoring robustness. Integrating RGB, multispectral, hyperspectral, and LiDAR data can capture multi-scale crop information under variable lighting, weather, and canopy occlusion [12,15]. Combining IoT sensor data with remote sensing data can improve early warning accuracy for diseases and pests, supporting data-driven decision-making in precision agriculture.

Domain adaptation and transfer learning will mitigate cross-environment generalization gaps. Models trained in specific regions often fail in new fields due to domain shift [15,65]. Short-term optimization of adversarial transfer learning and style normalization can improve model adaptability across different crops, regions, and growth stages, reducing reliance on large labeled datasets [66].

### 5.2. Long-Term Research Opportunities

In the long term, research will focus on foundational innovation and systematic integration, promoting AI from single-point applications to full-chain intelligent solutions. Key directions include foundation models, large-scale datasets, multimodal phenotyping platforms, and cross-domain adaptive systems.

Agricultural foundation models will become a core research direction. Integrating large language models (LLMs) and vision foundation models can unify visual recognition, agronomic reasoning, and decision-making [3,25]. These models can handle multi-tasks such as disease diagnosis, pest identification, yield prediction, and growth analysis, reducing the cost of developing task-specific models and improving cross-task generalization [67].

Large-scale, multi-environment agricultural datasets are fundamental for robust AI models. Current datasets such as PlantVillage, IP102, and DLCPD-25 are limited in diversity and regional coverage [12]. Long-term construction of multi-crop, multi-region, multi-season datasets covering diseases, pests, and nutrient stress will address data imbalance and scarcity, providing standardized benchmarks for model training and evaluation [68].

Multimodal phenotyping platforms will evolve into integrated monitoring systems. Combining UAV swarms, ground robots, and fixed sensor networks can achieve continuous, high-throughput monitoring across entire fields [28]. Integrating hyperspectral imaging and 3D reconstruction technology can capture subtle stress symptoms and structural traits, supporting early stress detection and growth assessment.

Cross-domain adaptive learning will break application boundaries. Combining self-supervised learning, federated learning, and domain adaptation can improve model generalization across different crops and regions [69]. Federated learning can realize collaborative modeling without data leakage, promoting cross-regional data sharing and model optimization.

### 5.3. Implementation Challenges

Despite promising directions, several practical challenges remain. Technological inclusion, capacity building, and policy standardization are key to promoting AI applications in crop production.

Technological inclusion and the digital divide are major barriers. Uneven digital infrastructure and high equipment costs limit AI promotion in underdeveloped regions and smallholder systems [1]. Developing low-cost, easy-to-operate AI devices and mobile applications can narrow the gap and improve technology accessibility.

Model interpretability and trustworthiness need improvement. Most deep learning models are “black boxes,” limiting farmer trust and adoption [3,4]. Applying SHAP and LIME interpretability methods can visualize decision-making processes, enhancing user acceptance.

Data standardization and regulatory systems are incomplete. Inconsistent data collection and annotation standards hinder cross-study integration [56,59]. Unclear data ownership and privacy risks restrict data sharing. Formulating unified agricultural AI standards and policies will support sustainable industry development.

In summary, future research should balance short-term optimization and long-term innovation, combining foundation models, large-scale datasets, multimodal platforms, domain adaptation, and lightweight edge systems. With technological progress and policy support, AI will play a core role in ensuring food security and promoting sustainable crop production.

## 6. Conclusions

Artificial intelligence technology has achieved multi-scenario technological breakthroughs in the agricultural field. From biotic stress monitoring to soil health management, from precision operations to supply chain optimization, and then to the construction of climate-resilient agriculture, AI technology has provided strong support for the precision, automation, and intelligence of agricultural production. Some applications have achieved high technical maturity and application effects. Four key technical pathways, including deep learning, sensor fusion, data-driven methods, and hybrid modeling, are constantly innovating, providing a solid technical foundation for the application of AI in agriculture.

However, the large-scale application of AI in the agricultural field still faces multiple challenges: regional bias, quality problems, and lack of standardization at the data level; insufficient model generalization, lightweight deployment, and interpretability at the technical level; digital divide, adaptability to smallholder farmers, and maintenance costs at the implementation level; and data privacy, algorithmic fairness, and imperfect regulatory systems at the ethical and policy level. These challenges are intertwined and require multi-dimensional and systematic solutions.

In the future, through the in-depth application of advanced AI models, multi-modal data fusion, and breakthroughs in lightweight and interpretability technologies, AI technology will achieve wider scenario expansion; through technological inclusion, capacity building, and improvement of policies and standards, the sustainable implementation of AI technology in the agricultural field will be promoted; and through in-depth alignment with sustainable goals such as carbon neutrality and resource recycling, AI will provide important support for the green and low-carbon transformation of agriculture. With the coordinated efforts of technological innovation and policy guarantees, artificial intelligence is expected to become a core driving force for ensuring food security and promoting sustainable agricultural development.

## 7. Material and Method

This systematic literature review originates from the following research questions: (1) How can artificial intelligence and machine learning methods enhance crop biotic stress monitoring and high-throughput phenotyping under field conditions? (2) What are the key challenges and technical gaps limiting the practical deployment of AI-driven agricultural systems?

The main objective of this study is to provide a comprehensive review of existing research that focuses on the use of artificial intelligence for the crop production process. This review aims to highlight how AI-driven techniques contribute to the advancement of precision agriculture by enabling efficient disease and pest detection, real-time crop health assessment, and data-driven crop production management.

**Figure 2:**
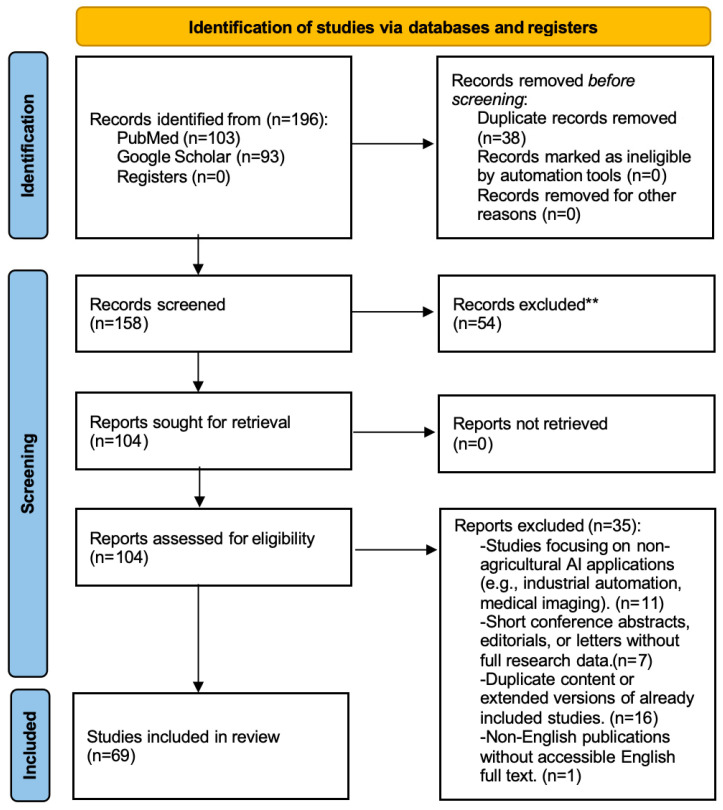
PRISMA flowchart. After the screening, 69 studies were selected to be included in the review. ** indicate how many records were excluded.

This type of work is referred to as a systematic review or survey paper. To achieve this, relevant studies were identified through a comprehensive search of journal articles, conference papers, book chapters, and existing reviews directly related to AI applications in crop production. This systematic review was performed using PRISMA (Figure 2) (Preferred Reporting Items for Systematic Reviews and Meta-Analyses) methodology (https://www.prisma-statement.org/prisma-2020-flow-diagram (accessed on 10 April 2026)).

This review focuses on papers published between January 2018 and May 2025 to highlight the most recent progress in AI-enabled crop phenotyping and stress detection. All selected papers relate to the use of AI, ML, and deep learning (DL) methods for biotic stress analysis and precision agriculture applications.

This work analyzed over 196 research papers and finally selected 83 papers for the systematic review. A specific focus is placed on papers that use machine learning, deep learning, transfer learning, self-supervised learning, hybrid models, and multimodal fusion in crop stress, disease, and pest recognition, plant phenotyping, and smart farming systems. These selected works included detailed comparisons of algorithms, model performance, datasets, and real-world applications. Details regarding the paper selection process are provided in the following subsection.

### 7.1. Search Techniques

All relevant research was retrieved from well-known databases and digital libraries, including ScienceDirect and Google Scholar. The literature search was conducted between 2018 and 2026. The following core keywords were used: artificial intelligence, machine learning, deep learning, convolutional neural network, crop, disease, pest, biotic stress, phenotyping, precision agriculture, hyperspectral, multispectral, UAV, remote sensing.

### 7.2. Eligibility Criteria

Studies were included if they met the following criteria:

(1) Peer-reviewed journal or conference papers published between 2018 and 2025;
(2) Focused on AI/ML/DL for crop disease, pest, or biotic stress detection;
(3) Applied field or experimental data for model validation;
(4) Included clear descriptions of model architectures, datasets, and performance metrics.

Studies were excluded if:

(1) Non-agricultural AI applications (e.g., industrial automation, medical imaging) (n = 7).
(2) Short conference abstracts, editorials, or letters without full research data (n = 4).
(3) Duplicate content or extended versions of already included studies (n = 9).
(4) Non-English publications without an accessible English full text (n = 1).

### 7.3. Selection Procedure

Following the PRISMA 2020 guidelines, a total of 196 records were initially identified. After removing duplicates and non-relevant entries, 158 records remained. Title and abstract screening excluded 54 irrelevant papers. Full-text evaluation excluded 35 papers. Finally, 69 peer-reviewed studies were retained for qualitative synthesis.

## Figures and Tables

**Table 1:** Main AI techniques for crop biotic stress monitoring and phenotyping.

Technique	Strengths	Limitations	Application Scenarios	References
Convolutional Neural Networks	Strong local feature extraction; robust to mild illumination changes; easy implementation	Poor global context; struggles with small pests; sensitive to complex backgrounds	Crop disease classification, leaf lesion detection	[2,5]
YOLO Series	Fast inference; real-time capability; suitable for dense objects	Lower accuracy for tiny pests; easy miss detection in clutter	Real-time pest detection, field monitoring	[8,22]
Vision Transformer	Captures global context; good for subtle symptoms	High computation; requires large datasets	Fine-grained disease diagnosis	[10]
Transfer Learning	Reduces data demand; fast training	Weak cross-domain generalization	Rare disease, new crop adaptation	[15]
Hybrid Models	Balanced local–global features; environment-robust	Complex; high cost	Multi-scale stress detection	[59]
Self-Supervised Learning	Low annotation dependency	Needs large unlabeled data	Data-scarce scenarios	[11]

## Data Availability

No new data were created or analyzed in this study. Data sharing is not applicable to this article.
